# How and why do bees buzz? Implications for buzz pollination

**DOI:** 10.1093/jxb/erab428

**Published:** 2021-09-19

**Authors:** Mario Vallejo-Marín

**Affiliations:** 1 Biological and Environmental Sciences, University of Stirling, Stirling FK9 4LA, UK; 2 University of Cambridge, UK

**Keywords:** Biomechanics, buzz pollination, communication, flight, Hymenoptera, pollen, pollination, poricidal flowers, thermoregulation, vibrations

## Abstract

Buzz pollination encompasses the evolutionary convergence of specialized floral morphologies and pollinator behaviour in which bees use vibrations (floral buzzes) to remove pollen. Floral buzzes are one of several types of vibrations produced by bees using their thoracic muscles. Here I review how bees can produce these different types of vibrations and discuss the implications of this mechanistic understanding for buzz pollination. I propose that bee buzzes can be categorized according to their mode of production and deployment into: (i) thermogenic, which generate heat with little mechanical vibration; (ii) flight buzzes which, combined with wing deployment and thoracic vibration, power flight; and (iii) non-flight buzzes in which the thorax vibrates but the wings remain mostly folded, and include floral, defence, mating, communication, and nest-building buzzes. I hypothesize that the characteristics of non-flight buzzes, including floral buzzes, can be modulated by bees via modification of the biomechanical properties of the thorax through activity of auxiliary muscles, changing the rate of activation of the indirect flight muscles, and modifying flower handling behaviours. Thus, bees should be able to fine-tune mechanical properties of their floral vibrations, including frequency and amplitude, depending on flower characteristics and pollen availability to optimize energy use and pollen collection.

## Teaser

The characteristics of buzz-pollinating vibrations, including frequency and amplitude, can be controlled through dynamic adjustment of the mechanical properties of the thorax, activation of indirect flight muscles, and flower handling.

One of the characteristics of modern biology is the breakdown of the boundaries which separate its subdivisions, and nowhere is this more fruitful than in behavioural studies where mechanics and physiology are integral to a more profound understanding.

J.W.S. Pringle (1957)

## Introduction

Written nearly 65 years ago, in the context of studying insect flight, the quote above also captures the enduring importance of multidisciplinary approaches to the study of the interactions between plants and their pollinators. Flowers and their visiting pollinators often interact intimately, and the properties of both floral structures and insect morphology, physiology, and behaviour become essential in determining the ecological and evolutionary outcomes of this interaction (Kevan and Lane, 1985; Chittka and Thomson, 2001; Reith *et al.*, 2006; Westerkamp and Classen-Bockhoff, 2007; Córdoba and Cocucci, 2011; Rands *et al.*, 2011; Whitney and Federle, 2013; Timerman and Barrett, 2018). The advantage of taking a multidisciplinary approach is illustrated in the study of buzz pollination, a unique form of pollination that has independently evolved multiple times in the evolutionary history of both flowering plants and bees (Buchmann, 1983; Cardinal *et al.*, 2018).

Flowers across many different families and involving >20 000 species have evolved tubular structures that keep pollen grains locked inside (Buchmann, 1983; De Luca and Vallejo-Marin, 2013; Russell *et al.*, 2017). In most cases, rigid tubular structures are formed by modified anthers or groups of anthers, which only release pollen via small apertures that range from tear-shaped slits to apical pores <100 μm in diameter (Buchmann, 1983; Puff *et al.*, 1995; Endress, 1996; Carrizo García *et al.*, 2008). In most of these poricidal flowers, pollen is the main or only available reward offered to floral visitors; that is, they are pollen flowers (Endress, 1994). Although floral visitors use diverse techniques to extract pollen from these flowers (Thorp, 2000), some species of bees have evolved the capacity to deploy powerful vibrations that cause pollen inside poricidal structures to be propelled out of the flower and onto the visitors’ body (Macior, 1964; Michener, 1962; Thorp and Estes, 1975; Buchmann *et al.*, 1977; King, 1993). These vibrations produced on flowers are sometimes called ‘sonication’, ‘vibratile pollen collection’, ‘pollination buzzes’, or ‘floral buzzes’ due to the audible ‘buzzing’ sound that is produced by the bee (De Luca and Vallejo-Marin, 2013; Russell *et al.*, 2016; Switzer and Combes, 2016). Although it was previously hypothesized that sound-induced vibrations (i.e. sonication) caused pollen to be ejected from flowers (DeTar *et al.*, 1968), it is now accepted that pollen release is associated with the mechanical shaking of anthers caused by direct contact of floral structures with the bee’s body, and thus here I use the term ‘floral buzzes’ or ‘vibratile pollen collection’ to refer to this type of bee behaviour (Vallejo-Marín, 2019).

Vibratile pollen collection has been recorded in >400 taxa belonging to 74 out of 508 recognized bee genera (table S1 in Cardinal *et al.*, 2018). Although the total number of bee species that can use vibratile pollen collection is unknown, the 74 genera documented so far include more than half of the 20 000 species of bees around the world (Cardinal *et al.*, 2018; Danforth *et al.*, 2019). Obtaining a more precise estimate of the number of species that can use vibrations to remove pollen from flowers will require a concerted effort to gather field observations of buzz-pollinating bees across a wide range of environments and taxonomic groups. Vibratile pollen collection has an idiosyncratic distribution among bee taxonomic groups (Buchmann, 1983; Thorp, 2000; Cardinal *et al.*, 2018). Currently there is no general explanation for why some bee species can buzz flowers while others, including honeybees (*Apis mellifera*) and many species in the families Andrenidae and Megachilidae, have never been observed buzzing flowers for pollen (King *et al.*, 1996; King and Buchmann, 2003; Cardinal *et al.*, 2018). Intriguingly, vibratile pollen collection is absent in the vast majority of other pollen-feeding insects, including most species of flies (Diptera) (but see Buchmann *et al.*, 1977). In pollen-gathering hoverflies (Syrphidae), individuals of some species are capable of producing vibrations of similar magnitude and characteristics to those of buzz-pollinating bees (Vallejo-Marín and Vallejo, 2021). This finding raises the possibility that the lack of more buzz-pollinating flies is explained not by biomechanical constraints in the production of the right type of vibrations, but perhaps by their life history and associated more modest pollen requirements compared with bees, in which larvae require the provision of large amounts of pollen (Woodcock *et al.*, 2014; Inouye *et al.*, 2015; Vallejo-Marín and Vallejo, 2021).

The floral buzzes produced by some bees during buzz pollination belong to one of several other types of behaviours that involve vibrations produced with bees’ thoracic muscles (Hrncir *et al.*, 2008a). Vibrations produced by rhythmic contraction of the main thoracic muscles in bees are associated with behaviours that range from flight to communication to foraging for pollen. Although there are many excellent studies analysing bee vibrations in specific behavioural contexts, including flight (Casey *et al.*, 1985; Dudley and Ellington, 1990), thermogenesis (Heinrich and Esch, 1994), communication (Hrncir *et al.*, 2005; Conrad and Ayasse, 2015), and pollination (King *et al.*, 1996; Switzer *et al.*, 2019), to date no previous work has attempted to jointly review how bees buzz across all these behaviours, and this is the main goal of the present study. Accordingly, in this review, I will synthesize current knowledge regarding the use of vibrations in different behavioural contexts in bees, focusing on their mechanism of production and the extent to which non-flight floral vibrations can be controlled by the bee. My goal is to provide a framework for futures studies on buzz pollination that incorporates knowledge of the mechanisms of vibration production for addressing hypotheses about bee–flower interactions.

## The mechanisms generating bee vibrations: thoracic power muscles

Bee vibrations are the product of rhythmic oscillatory contractions of muscles located in the thorax (Pringle, 1957; Dudley and Ellington, 1990; Dickinson, 2006). In bees, flies, and other insects, two sets of thoracic power muscles occupy most of the thorax and drive its oscillatory movement (Snodgrass, 1935; Marden, 1987; Dickinson, 2006). These muscles are responsible for providing the power required to flap wings as well as generating all other types of bee buzzes. Because of their role in powering flight, the thoracic power muscles have long been best studied in this context (Pringle, 1957; Dickinson and Tu, 1997; Dudley, 2000).

### Indirect flight mechanism

The function of the muscles that power flight and generate other types of buzzes is best understood by placing them in their anatomical and physiological context. Wing movement in bees (and flies, beetles, and other insects) is caused indirectly by changing the conformation of the thorax (Dickinson *et al.*, 1998; Klowden, 2013). Wings are attached to hardened plates in the exoskeleton and not directly to the muscles that cause wings to beat. When the indirect flight muscles contract, the thorax deforms and a hinge-type attachment of the wings to the thorax then causes the wings to move upward and downwards as the thorax changes shape (Dickinson and Tu, 1997; Klowden, 2013).

**Fig. 1.**
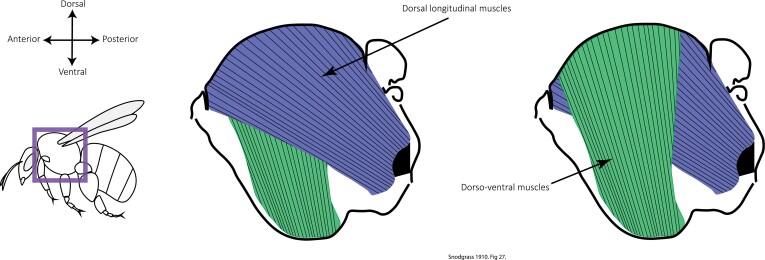
Simplified diagram of the indirect flight muscles that power the oscillations produced by the thorax. A pair of dorsal longitudinal muscles (DL; purple) are flanked by a pair of dorso-ventral muscles (DV; green) located towards the centre of the thorax. Muscle diagrams modified from Snodgrass (1910).

Indirect flight muscles are grouped into two major types (Pringle, 1957; Dickinson and Tu, 1997; Klowden, 2013) that are discussed in turn. (i) Dorso-ventral (DV) muscles. This pair of muscles are attached from the tergum (dorsal side) to sternum (ventral side) of the insect’s thorax (Fig. 1). The contraction of this muscle pair causes deformation of the thorax by pulling the dorsal part of the thorax (tergum) down. During flight, pulling the tergum down indirectly causes the wings to rise though an elegant hinge mechanism that connects the wings and the exoskeletal plates of the thorax (tergites) (Dickinson, 2006). The contraction of the DV muscles during flight therefore causes the upstroke of the wings. (ii) Dorsal-longitudinal (DL) muscles. The DL muscles are flanked by the DV muscles (Fig. 1). The DL muscle pair attaches longitudinally in the thorax on the wing-bearing thoracic segment (Pringle, 1957). When the DL muscles contract, they shorten the wing-bearing thoracic segment and cause the tergum to arch and elevate. The thorax deformation, aided by the wing hinge mechanism, then causes the wings to move downwards, producing a downstroke (Dickinson, 2006; Klowden, 2013). Because of the indirect effect of these muscles on the movement of the wings via thoracic deformations rather than direct attachment to the wings, the DL and DV muscles are known as indirect flight muscles. These muscles are capable of generating considerable power, and it is estimated that DV muscles in bumblebees can produce ≥100 W kg^−1^ during flight (Josephson, 1997).

In addition to the indirect flight muscles, there are a series of other muscles, including the accessory indirect muscles that control flight (Pringle, 1957; Nachtigall and Wilson, 1967; Dickinson and Tu, 1997; Dickinson *et al.*, 1998; Dickinson, 2006). These accessory muscles insert into the thorax and modify its movement and mechanical conformation (Pringle, 1957; Dickinson *et al.*, 1998; Klowden, 2013). For example, the pleurosternal [thorax muscles that run from the sternum (ventral side) to the pleuron (lateral side) of the thorax] and pleurotergal muscles [thorax muscles that run from the tergum (dorsal side) to the pleuron (lateral side) of the thorax] modulate the power output and the nature of the wingbeat by changing the orientation of the thoracic plates and the resonance of the thorax (Klowden, 2013). Other axillary muscles (muscles that insert in hardened axillary thoracic plates or sclerites) affect wing supination and wing flexion against the body when wings are at rest (for illustrations of these muscles, see Klowden, 2013; and for diagrams of bee anatomy, see Snodgrass, 1935).

### Physiology and metabolism of thoracic muscles

The DL and DV thoracic muscles responsible for powering flight are a special type of muscle called asynchronous. Asynchronous muscles have evolved in different insect groups (Deora *et al.*, 2017) and represent a major innovation in the evolutionary history of insects (Darveau *et al.*, 2005). These asynchronous muscles (also called myogenic or fibrillar) can contract at a faster rate than the rate of motor neuron impulses they receive via a phenomenon called stretch activation (Pringle, 1949; Dickinson and Tu, 1997; Josephson *et al.*, 2000). The contraction of one set of muscles causes deformation of the thorax which stretches the opposite set of muscles. The process of stretching then causes muscles to contract, triggering a cycle of stretch-activated contractions for a few cycles until the next neuron action potential is received (Josephson *et al.*, 2000). Stretch activation decouples neural muscle activation and contraction, and allows thoracic oscillations at a relatively high frequency (Darveau *et al.*, 2005), and a much higher rate than the frequency of neural stimulation (Heinrich, 1993).

Activation of the indirect flight muscles requires a significant amount of energy (Dudley, 2000). To contract the thoracic muscles and flap the wings at magnitude and frequencies high enough to enable flight, bees must convert chemical energy into mechanical energy. In insects, this energy conversion is very inefficient, with ~10–20% of chemical energy used for flight being converted to mechanical energy, and the rest dissipated as heat (Dudley, 2000; Klowden, 2013). It is therefore not surprising that flying insects have one of the highest rates of aerobic metabolism in animals (Darveau *et al.*, 2005). The flight metabolism in species with high wingbeat frequency is aerobic, involving mostly carbohydrate oxidation (Klowden, 2013). In bees, flight is fuelled almost exclusively by carbohydrates, and has an equivalent rate of O_2_ consumption and CO_2_ production (Darveau *et al.*, 2005). During flight in bees, >90% of the oxygen consumed is accounted for by oxidative metabolism in the flight muscles (Darveau *et al.*, 2005). The metabolic requirements for activation of the indirect flight muscles during flight is expected to vary with insect size as well as with wingbeat frequency.

In bees, metabolic rate scales positively with body mass, but mass-specific metabolic rate (per gram) scales negatively with body mass (Darveau *et al.*, 2005; Niven and Scharlemann, 2005; Grula *et al.*, 2021). The body mass effect on metabolism during hovering flight occurs through scaling of wing form and wing loading. In turn, these determine wingbeat frequency and therefore metabolic rate. In aerobic muscles, operating frequency is the primary determinant of power output (Pennycuick and Rezende, 1984) and, in bees, as expected, wingbeat frequency is positively correlated with metabolic rate (Darveau *et al.*, 2005). Thus metabolic rate scales positively with wingbeat frequency but negatively with mass, wing length, wing area, and wing loading (Darveau *et al.*, 2005). The various routes in which form and function are related in flying insects is thus a good reminder of the importance of taking an integrative approach for linking morphology, physiology, and function.

## A classification of the different types of bee vibrations

**Fig. 2.**
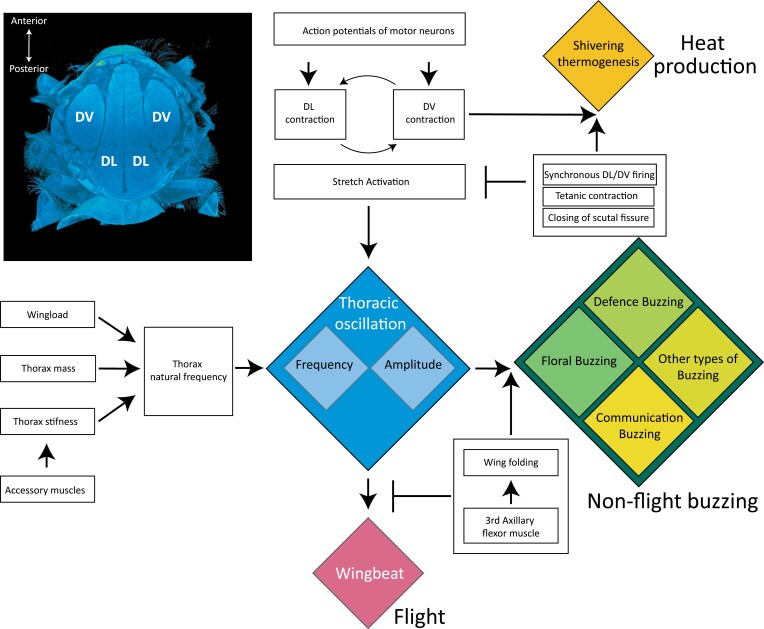
Overview of the production of vibrations across different behavioural contexts and their hypothesized control and modulation mechanisms. Ultimately, all thoracic oscillations are driven by the DL/DV thoracic muscle contractions. Both neural and mechanical controls can prevent the stretch activation response, resulting in shivering thermogenesis (gold diamond). When stretch activation occurs, thoracic deformation triggers thoracic oscillations (blue diamond). Thoracic oscillations are modulated by the material and mechanical properties of the thorax and by the contraction of accessory muscles. Wingbeat can be allowed or prevented by activation of accessory muscles that control wing folding (pink diamond). In the absence of wingbeat, thoracic oscillations yield non-flight vibrations deployed in different behavioural contexts including communication, defence, and floral buzzing (green diamond). Inset: X-ray microcomputed tomography (X-ray μCT) of the dorsal view of the thorax of a worker of *Bombus terrestris* ssp. *audax*. The image shows a longitudinal section approximately halfway through the thorax and illustrates the dorsal longitudinal muscles (DL) flanked by the dorso-ventral muscles (DV). X-ray μCT image by Sarah Aldridge.

Ultimately all vibrations produced by bees trace back their proximate origin to the contraction of the energy-demanding indirect flight muscles described above. Although usually considered separately, vibrations produced in different behavioural contexts can be considered in a single conceptual framework (Fig. 2). Using this perspective helps to understand the potential control and modulation mechanisms that allow some bees to quickly shift and deploy different types of vibrations that rely on the activation of similar sets of muscles. Below, I suggest a classification of bee buzzes into three main types: (i) thermogenic activity used to produce heat with minimal to no thoracic oscillation; (ii) thoracic oscillations that drive wingbeat and enable flight; and (iii) non-flight vibrations in which the thorax oscillates but the wings remain undeployed, and which produce air- or substrate-borne vibrations that are associated with communication, defence, and vibratile pollen collection. Next, I provide a brief overview of these three main types of bee vibrations and their hypothesized control and modulation mechanisms.

## Non-flight or shivering thermogenesis

Some bees engage in non-flight thoracic muscle activity to generate heat, a phenomenon known as shivering thermogenesis or non-flight thermogenesis (Heinrich, 1993; Potts *et al.*, 2018). In bees, shivering thermogenesis occurs when the bee is either walking or stationary, and does not result in perceptible airborne sound (Heinrich, 1993). The effect of non-flight thermogenesis is remarkable as, through this behaviour, the thorax temperature can increase >30 °C (from ~6 °C to 37 °C) in <20 min (Heinrich, 1993). This thermogenic behaviour is important at the individual bee level because it produces heat necessary to initiate flight (Heinrich and Kammer, 1973). The capacity to produce these warm-up buzzes enables bees to initiate flight at relatively low ambient temperatures. In addition, in social bees, including *Apis* and *Bombus*, non-flight thermogenesis is used to regulate the temperature of the colony (Heinrich, 1972; Kleinhenz *et al.*, 2003; Livesey *et al.*, 2019).

Heat production during non-flight thermogenesis is achieved exclusively by action of the thoracic muscles (Heinrich, 1993). However, a very peculiar characteristic of non-flight thermogenesis is that although it involves contraction of the indirect flight muscles (Esch *et al.*, 1991), there is little to no oscillation of the thorax (Surholt *et al.*, 1990). Warm up thoracic muscle activity is therefore relatively motionless.

Motionless activation of the indirect flight muscles in non-flight thermogenesis can be achieved through three mechanisms (Heinrich, 1993). (i) Isometric contraction of sets of opposing muscles. During warm up, the DL and DV motor neurons fire synchronously, which might reduce the mechanical action of the muscles as they are contracting in a synchronous fashion against each other (Heinrich, 1993). (ii) Mechanical stop/prevention of stretch activation. During warm-up buzzing, the DL muscles are activated at a greater frequency than the DV muscles. The higher activation of the DL muscles might stretch the DV muscles and cause the scutellar fissure in the thorax to close (for a diagram of the bee thorax showing the scuttelar fissure, see King *et al.*, 1996). This might act as a mechanical stop that prevents movement of the muscle, mechanical oscillations, and the stretch activation response (Esch and Goller, 1991). Thus, during thermogenesis, and in contrast to flight, contraction of one set of indirect flight muscles (e.g. DL) does not result in stretching of the opposing set (e.g. DV) and therefore there is no oscillation of the thorax or the wings during shivering thermogenesis (Surholt *et al.*, 1990; Heinrich, 1993). (iii) Tetanic contraction. Activation of the indirect flight muscles at frequencies >15 Hz can cause tetanus of the muscle (Heinrich, 1993). During non-flight thermogenesis in bumblebees, the DL muscles are activated at frequencies of up to 40 Hz, resulting in muscle tetanus that generates tension but little motion. The action of these three mechanisms helps to explain how muscle activity and thorax oscillation can be decoupled from one another during non-flight thermogenesis (Fig. 2).

The rate of heat production during non-flight thermogenesis is thought to be under the control of the bee. In honeybees, there is a 1:1 correspondence in action potentials and muscle contraction during shivering (Esch, 1964; cited in Heinrich, 1993). The frequency of action potentials and the amount of contraction of the muscle being activated are positively related, and heat production is thus a direct function of action potential frequency (Heinrich, 1993). Since bees that are not in flight can activate their indirect flight muscles at a wide range of frequencies (Heinrich, 1993), it is expected that heat production should be under neural control.

## Thoracic oscillations during flight

The transition from thermogenic to flight-associated thoracic vibrations involves changes in the rate of DV action potentials which shorten the DV muscle and start stretch activation of the DL muscles (Heinrich, 1993). The stretch activation response then causes thoracic deformation and, with wings deployed, results in wingbeats (Dickinson *et al.*, 1998). In flight, the stretch response is maintained by action potentials firing approximately every 10 muscle contractions (Heinrich, 1993).

Wingbeat frequency increases with the frequency of action potentials (Esch and Bastian, 1968; Bastian and Esch, 1970). However, during flight, stretch activation of the thoracic muscles generally occurs at the natural frequency of the oscillating system (Hrncir *et al.*, 2008a), suggesting that the system behaves as a resonant system (Jankauski, 2020). In a bee, the natural frequency of the oscillating system is determined by several factors including the elasticity of the thorax capsule, muscle tension, and inertial load (corresponding to the mass distribution along the wing length) (Darveau *et al.*, 2005; Hrncir *et al.*, 2008a). Empirical studies show that adding mass to the thorax or clipping the wings (which changes both mass and moment of inertia) changes the frequency of flight vibrations as expected for a resonant system (Hrncir *et al.*, 2008a). It is thought that by driving wingbeat frequencies at the natural frequency of the thorax, bees can optimize power output for a given input of energy.

### Correlates of wingbeat frequency

Wingbeat frequency is negatively associated with body size across insect orders (Greenewalt, 1960; Dudley, 2000; Deora *et al.*, 2017), and across multiple bee species (Corbet and Huang, 2014; De Luca *et al.*, 2019). Darveau *et al.* (2005) conducted a study on orchid bees (Euglossini) and used phylogenetically independent contrasts to look at correlations between wingbeat frequency and body mass, wing morphology (length and area), and energetic costs. They found that wingbeat frequency declines with body mass, wing area, and wing length (Casey *et al.*, 1985). When analysed individually, wing area and wing length explained a higher proportion of wingbeat variation than body mass (Darveau *et al.*, 2005). However, because wing area and body mass are positively correlated, Darveau and colleagues also analysed the residuals of wing loading after accounting for body mass, revealing a positive association between wing loading and wingbeat frequency, both in their dataset and during re-analysis of a previous study on homopterous insects (Byrne *et al.*, 1988). From the considerations above, two general inferences can be made: first, in flight, wingbeat frequency can be driven by changes in the activation rate of the indirect flight muscles in response to different firing rates of motor neurons. Second, thoracic oscillations of the thorax during flight are generally driven at the resonant frequency of the flying insect as dictated by the natural frequency of the system, which depends on body and wing size, properties of the thoracic capsule, and tension of accessory muscles in the thorax (Fig. 2).

## Non-flight thoracic vibrations

Non-flight thoracic vibrations that are not associated with thermogenesis encompass oscillations produced during a wide range of behavioural contexts, but which share two properties: (i) the thorax of the bee oscillates in response to deformation of the cuticle driven by contractions of the indirect flight muscles; and (ii) wings remain undeployed; that is, folded in a resting position above the thorax and abdomen of the bee, limiting their displacement and becoming decoupled from the indirect flight mechanism (King, 1993). Folding of the wings is achieved in a clutch-like manner by contraction of the flexor muscle or the third axillary muscle, which causes wing rotation and folding through a complex set of interactions with skeletal plates (Heinrich, 1993; Pringle, 1957). Although some bees and wasps use vibrations to compact soils within nests (Spangler, 1973; King *et al.*, 1996) or as cues for localization (Larsen *et al.*, 1986), most non-flight vibrations are produced in the contexts of communication, defence, or during buzz pollination. Below I discuss these different types of non-flight vibrations in bees.

### Communication buzzes

Thoracic vibrations are involved in communication in some species of social bees (Hrncir *et al.*, 2005). In honeybees (Apidae: Apini), workers produce vibrations, known as hissing and piping, that are thought to aid worker–worker communication in different behavioural contexts including during the lift off of a swarm (Hrncir *et al.*, 2011; Seeley and Tautz, 2001). Piping calls are characterized by a higher fundamental frequency than in flight, as well as by the presence of harmonics, which together are perceived by humans as a high-pitched sound (Seeley and Tautz, 2001). When the colony is disturbed either by mechanical jolts or by the presence of potential predators, worker piping can trigger the simultaneous hissing of other bees in the colony, resulting in a coordinated response of the entire colony (Hrncir *et al.*, 2005). The spectral properties of hissing and piping vibrations are diverse but, unlike the pure tones that characterize flight, can include broad frequency spectra. Piping calls can be produced with wings folded or with wings set apart, and within a single piping call the frequency is modulated from low to high (Seeley and Tautz, 2001). Hissing calls are broadband with predominantly high frequencies, and are sometimes accompanied by low-amplitude wing movement and thus can have both an acoustic and a visual component (Hrncir *et al.*, 2005). Honeybee queens also vibrate to produce a signal type known as tooting, which exhibits both frequency and amplitude modulation (Seeley and Tautz, 2001). In some stingless bees (Apidae: Meliponini), vibrations are also produced when foragers communicate inside the nest (Hrncir *et al.*, 2005, 2006). Some stingless bees (*Melipona* spp.) can apparently use the airborne sounds associated with these vibrations to communicate with nestmates about floral resources (Hrncir *et al.*, 2006, 2008*b*).

### Mating buzzes

Field observations suggest that vibrations are used by multiple bee species during mating, but this has been documented in detail in only a few cases (Buchmann, 1983; Alcock and Buchmann, 1985; Conrad and Ayasse, 2015). For example, males of *Panurgus* and *Colletes* produce buzzing sounds during copulation (Larsen *et al.*, 1986; Tengö *et al.*, 1988) and, in *Centris pallida*, males appear to use vibratile signals during copulation to reduce the receptivity of females to subsequent mating (Alcock and Buchmann, 1985). Moreover, some species of *Osmia* (Megachilidae) use vibrations during courtship and mating (Conrad and Ayasse, 2015). Here, males produce vibrations that influence the likelihood of successful mating. These mating vibrations can be population specific and provide an example of how thoracic vibrations could play a role as a mechanism of mate recognition and reproductive isolation among populations. Further detailed studies of the extent to which other bee species use non-flight vibrations during mating, and quantitative analyses of the potential variation in vibration characteristics, will help in illuminating a relatively understudied mode of insect vibrational communication with a direct link to reproductive success.

### Defence buzzes

Non-flight thoracic vibrations are deployed by some bees (and some wasps and flies) as a warning or defensive signal (Heinrich, 1993; Josephson and Ellington, 1997; Kirchner and Röschard, 1999). Defence vibrations are generally produced with wings folded and can be heard by humans as a high-pitched buzzing sound. During defence buzzing in bumblebees, the DL muscles are activated at a higher frequency than the DV muscles, resulting in the stretch activation response and oscillatory deformation of the thorax (Heinrich, 1993). Josephson and Ellington (1997) analysed the thorax contraction frequency and strain of DV muscle during flight and defence (escape) in *Bombus terrestris*. Their results suggest that defence thoracic contractions occur at approximately twice the frequency (200–300 Hz) as thoracic contractions during tethered flight (100–150 Hz). The strain of muscles (percentage muscle elongation) during defence buzzing has a similar range to that during flight (1–3%). However, comparison of thorax contraction frequency and strain in tethered flight shows that frequency and strain are positively associated (Josephson and Ellington, 1997). Separate studies in *B. terrestris* confirm that defence buzzes have a statistically significant higher fundamental frequency that flight buzzes (King and Buchmann, 2003; Pritchard and Vallejo-Marín, 2020). This increase in the frequency of defence buzzes compared with flight buzzes has also been documented in two species of *Xylocopa* (King and Buchmann, 2003) and in *Melipona seminigra* (Hrncir *et al.*, 2008a). Analyses of the comparative magnitude of defence and flight vibrations show mixed results. Some studies show that both the acoustic relative amplitude (dB) in *B. terrestris/B. lucorum* and *B. pascuorum*, and the vibration amplitude displacement (μm) in *B. terrestris* and *Xylocopa* spp. are lower in defence than in flight (King and Buchmann, 2003; De Luca *et al.*, 2014). In contrast, a study in *B. terrestris* using laser vibrometry shows that thoracic vibration amplitude (measured as either velocity, acceleration, or displacement peak amplitude) of defence buzzes is higher (Pritchard and Vallejo-Marín, 2020). Similarly, comparison of tethered flight and defence (annoyance) buzzing in *M. seminigra* shows a 2- to 5-fold increase in peak-to-peak displacement and velocity amplitude of defence vibrations (Hrncir *et al.*, 2008a).

### Floral buzzes

The acoustic–mechanical properties of floral buzzes have been characterized across a number of bee species visiting plants in the families Actinidiaceae, Ericaceae, Boraginaceae, Melastomataceae, Primulaceae, Orobanchaceae, and Solanaceae, among others (e.g. Macior, 1964; Thorp and Estes, 1975; Buchmann *et al.*, 1977; Corbet *et al.*, 1988; King and Buchmann, 1995, 1996; Corbet and Huang, 2014; Switzer and Combes, 2016; Arroyo-Correa *et al.*, 2019; De Luca *et al.*, 2019). The most widely assessed property of floral buzzes is frequency (usually the fundamental or dominant frequency), in part because, unlike vibration amplitude, it can be accurately determined in laboratory and field studies with easy to use acoustic recorders (De Luca *et al.*, 2018). Floral buzzes have a higher fundamental frequency than vibrations associated with flight across several species in the families Andrenidae, Apidae, Colletidae, Megachilidae, and Halictidae (Corbet *et al.*, 1988; Harder and Barclay, 1994; King, 1993; King *et al.*, 1996; Burkart *et al.*, 2011; Cane, 2014; Corbet and Huang, 2014; De Luca *et al.*, 2019), and it is very likely that this is a general pattern across all buzz-pollinating bees. However, the increase in frequency during floral vibrations relative to flight vibrations varies. In large bees, such as *Bombus*, *Centris*, *Eulaema*, and *Xylocopa*, the floral:flight frequency ratio is between 1.3:1 and 2.5:1, while in smaller bees including *Dialictus*, *Exomalopsis*, *Nomia*, *Agapostemon*, *Augochloropsis*, *Pseudoaugochlora*, *Melipona*, and others it is between 1:1 and 2:1 (Macior, 1968; Harder and Barclay, 1994; Burkart *et al.*, 2011; Corbet and Huang, 2014; De Luca *et al.*, 2019). Indeed, some evidence suggests that there is a positive relationship between bee size and the floral:flight frequency ratio (Burkart *et al.*, 2011; De Luca *et al.*, 2019). Although floral and defence buzzes are produced by similar non-flight thoracic vibrations, the few studies that have directly compared them suggest that their mechanical properties also differ. In *B. terrestris*, floral vibrations measured at the bee’s thorax using laser vibrometry have higher fundamental frequency and peak amplitude (displacement, velocity, and acceleration) than defence buzzes (Pritchard and Vallejo-Marín, 2020). In the next section, I discuss some potential mechanisms that might help explain differences in the properties of non-flight vibrations produced by bees.

## Mechanisms determining the properties of non-flight bee vibrations

The natural frequency of the thorax plays an important role in setting the frequency of wingbeat (Greenewalt, 1960; Dudley, 2000), and changes in resonant properties are probably one of the principal determinants of the operational frequency of the thorax during non-flight vibrations as well (King, 1993). Although the frequency of nerve signals to the indirect flight muscles is positively correlated with the amplitude and frequency of wingbeats, it is thought that it does not strongly influence the frequency of thoracic oscillations (Machin and Pringle, 1959; Nachtigall and Wilson, 1967). Nerve impulses to the indirect flight muscles are responsible of activating and maintaining the stretch response of the DV and DL muscles, but the frequency of muscular contraction and thorax oscillation appears to be mostly determined by the resonant properties of the thorax (Greenewalt, 1960; Nachtigall and Wilson, 1967; Dickinson and Tu, 1997; Dudley, 2000). Resonance occurs when the rate of muscle contraction of the indirect flight muscles (the driving force of oscillations) coincides with the natural frequency of the system (i.e. the bee or coupled bee–flower). Under these conditions, the amplitude of the oscillations is expected to increase to an extent determined by the damping properties of the system (Denny, 2015). The higher frequency of non-flight vibrations compared with thoracic vibrations that occur during flight has been explained by an increase in the natural frequency of the thorax (King, 1993; King *et al.*, 1996; Seeley and Tautz, 2001; King and Buchmann, 2003) achieved when folding and decoupling the wings, which reduces inertial load on the oscillating system (Esch and Wilson, 1967). In addition, the thorax can be stiffened via increased tension associated with wing adduction and with activity of the accessory muscles in the thorax (King, 1993; Dudley, 2000; Seeley and Tautz, 2001; Jankauski, 2020). The actions of accessory muscles that fold the wings and cause changes in thorax stiffness are under the neural control of the insect (Nachtigall and Wilson, 1967; Dickinson and Tu, 1997) and thus could be used as a modulation mechanism to change vibration frequency.

A non-mutually exclusive possibility for control of thoracic oscillations is that the muscle contractions of the thorax during non-flight vibrations occur at a frequency higher than the natural frequency of the system (i.e. they are driven oscillations), with the concomitant increase in energy required to maintain these forced oscillations (King *et al.*, 1996; Dudley, 2000; King and Buchmann, 2003; Hrncir *et al.*, 2008a). This hypothesis is considered unlikely to be a main driver of vibration frequency due to the indirect action of power flight muscles (Machin and Pringle, 1959) as well as to the steep increase in energetic requirements to drive the system above its natural frequency (Dudley, 2000). In a study of *Melipona seminigra*, Hrncir *et al.* (2008a) explored the hypothesis that non-flight vibrations are produced above thorax resonance (i.e. that they are driven oscillations). It is expected that during driven oscillations, the bee’s thorax should vibrate at the frequency of the rate of muscle contraction, but that the vibration rate should immediately decay to the natural frequency as soon as the driving force stops (Bennet-Clark, 1999; Hrncir *et al.*, 2008a). In contrast, a system vibrating at its natural frequency should not change in frequency during the build-up and decay phases (Hrncir *et al.*, 2008a). By studying changes in frequency during the build-up and decay periods of three types of vibrations (stationary flight, annoyance buzzes, and forager communication vibrations), Hrncir *et al.* (2008a) determined that while flight vibrations occur at the natural frequency, non-flight vibrations do not. They conclude that resonance does not play a major role in non-flight vibrations. Interestingly, wing removal and experimental mass loading in *M. seminigra* increase the dominant frequency of flight wingbeats, supporting the resonance hypothesis. In contrast, the effects of wing clipping, and mass loading do not change the frequency of non-flight vibrations (Hrncir *et al.*, 2008a). However, mass loading reduces the amplitude of non-flight vibrations, which the authors explain as a consequence of constant force during non-flight vibrations. Based on these observations, Hrncir and colleagues suggest that the higher main frequency of non-flight vibrations is controlled by increasing the rate of neuron excitation of the indirect flight muscles, rather than changes to the natural frequency of the thorax alone.

**Fig. 3.**
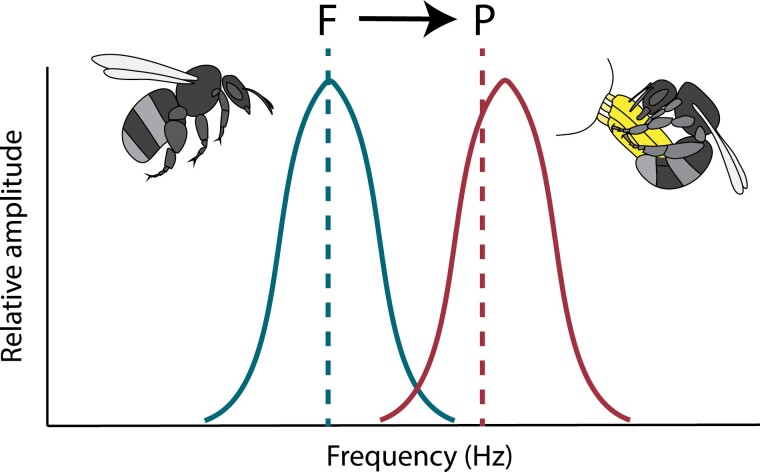
Hypothesized change in the relationship between natural frequency and thorax oscillation frequency between flight (blue; F) and floral (red; P) vibrations. The dashed line represents the natural frequency of the system at which resonance is expected to occur. The natural frequency of the system is influenced by the mass and inertial loading of the bee or coupled bee–flower system. Changes in wing deployment and stiffness of accessory muscles in the thorax cause a shift in natural frequency during flight (F; blue dashed line) and floral (P; red dashed line) vibrations. The solid lines represent the distribution of frequencies generated by individual bees through the contraction of the indirect flight muscles under the two different behaviours. Notice that while flight vibrations closely match the natural frequency, floral vibrations could be driven at a higher frequency than the corresponding natural frequency of the thorax.

The resonance and driven oscillation hypotheses are not mutually exclusive, and both might play a role in explaining the mechanism producing high-frequency vibrations during non-flight behaviours. On one hand, wing folding, activation of control muscles, and muscle stiffening probably change the natural frequency of the thorax, leading to a new resonant frequency compared with that under flight (Fig. 3) (Esch and Wilson, 1967; Nachtigall and Wilson, 1967; Dickinson *et al.*, 1998; Gau *et al.*, 2021). Thus, as in flight, the frequency of floral vibrations is likely to be primarily determined by the mechanical properties of the thorax, including its resonant frequency (Nachtigall and Wilson, 1967; Dudley, 2000). On the other hand, increased rates of neuron firing changing indirect flight muscle contraction may be a secondary mechanism driving the thorax to vibrate above this new resonant frequency (Heide, 1974; King *et al.*, 1996; Dickinson *et al.*, 1998) (Fig. 3). Less is known about the potential for bees to control the amplitude of thoracic vibrations. During flight, experiments on bees show that they can increase wingbeat amplitude in response to carrying heavier loads, producing more translational force (Combes *et al.*, 2020), which may be partially due to changes in the amplitude of thoracic oscillations. In the indirect flight muscles of flies, changes in the frequency of electric spikes in muscles are thought to affect power output (Dickinson and Tu, 1997). Although further work is required to investigate these and other hypotheses of the control of thoracic vibrations, bees are likely to possess mechanisms to modulate the frequency and amplitude properties of non-flight vibrations, including floral vibrations.

## Implications for buzz pollination

**Fig. 4.**
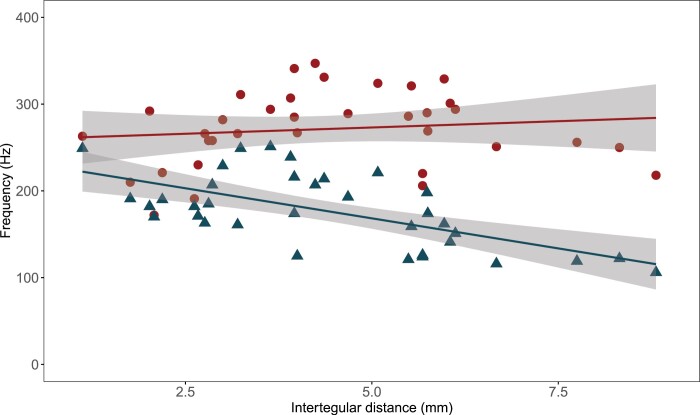
Relationship between frequency (Hz) and bee size (intertegular distance, mm) for both floral vibrations (red circles) and wingbeat (blue triangles) for 35 species of bees sampled in tropical and temperate communities in the Americas. Each bee species is represented by a pair of symbols (one circle and one triangle). The lines and grey regions represent a linear model and its associated 95% confidence interval calculated with the function geom_smooth in R (R Development Core Team, 2021), fitted separately for flight and floral buzzes. The Pearson’s correlation coefficient (95% confidence interval) for flower buzzes is ρ=0.126 (–0.216, 0.441) and for wingbeat is ρ= –0.628 (–0.795, –0.374). Data from Burkart *et al.* (2011) and De Luca *et al.* (2019).

Buzz-pollinating bees extract pollen from flowers with enormously different sizes, shapes, material properties, and, presumably, different vibrational properties (Buchmann, 1985; Vallejo-Marín, 2019). Flexibility in the capacity to generate and apply floral vibrations could give bees the behavioural and biomechanical tools to exploit diverse, pollen-rich plants. Mechanisms including variation in the degree of wing deployment, changes in thoracic stiffness through accessory muscles, and rate of neural activation of indirect flight muscles are potential processes that could allow bees to modulate the amplitude and frequency of floral vibrations. For example, there is a strong correlation between wingbeat frequency and body/wing size (Darveau *et al.*, 2005), presumably because during flight wingbeats occur at the natural frequency of the system, which is strongly affected by these morphological characteristics (Jankauski, 2020). In contrast, if floral vibrations can be produced at higher frequencies than the frequency dictated by the resonance of the bee or the bee–flower system, then bees of different sizes and morphologies might be able to produce similarly high-frequency floral vibrations. Consistent with this idea, the frequency of floral vibrations does not scale with body size as strongly as wingbeat frequency, as has been shown in studies of bee communities in temperate and tropical regions in the Americas (Burkart *et al.*, 2011; De Luca *et al.*, 2019; Rosi-Denadai *et al.*, 2020) (Fig. 4). Producing vibrations at maximum frequency regardless of bee size might be beneficial to increase the rate of pollen removed using vibrations (Corbet and Huang, 2014; Switzer *et al.*, 2019). For a given maximum displacement of the thorax, higher frequency vibrations should result in larger velocity and acceleration amplitudes, which in turn are associated with more pollen released from buzz-pollinated flowers (Buchmann and Hurley, 1978; Harder and Barclay, 1994; King and Buchmann, 1996; De Luca *et al.*, 2013; Corbet and Huang, 2014; Vallejo-Marín, 2019; Rosi-Denadai *et al.*, 2020; Kemp and Vallejo-Marin, 2021).

**Fig. 5.**
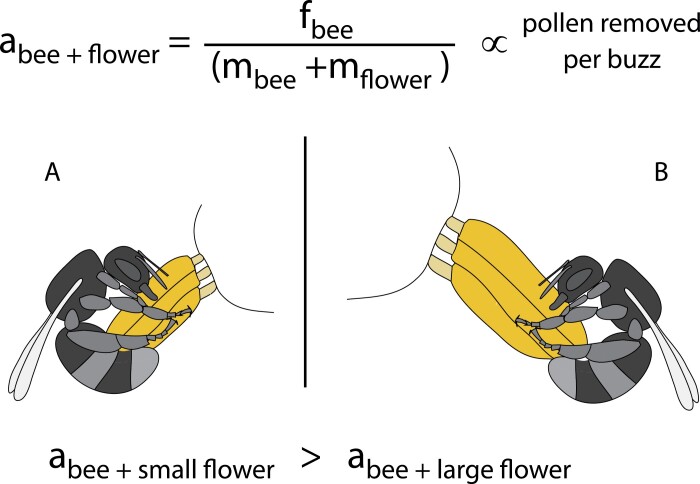
Hypothesized effect of flower (stamen) mass on the accelerations achieved by an individual bee. A bee of certain mass (m_bee_) and characteristics should be able to generate a given maximum force (f_bee_) using the acceleration (a_bee_) of its thoracic power muscles (f_bee_=m_bee_×a_bee_). When the bee applies these vibrations to the flower, the mass of the flower (m_flower_) is added to the bee–flower coupled system, decreasing the acceleration proportionally to the mass of the flower. A bee visiting a flower with relatively smaller stamens (A) should achieve higher anther accelerations than the same bee visiting a flower with larger stamens (B). The positive relationship between anther acceleration and pollen release then predicts that, all else being equal, the rate of pollen removal should also decrease with flower mass.

Because of the functional connection with pollen release, increasing velocity/acceleration amplitudes of the vibrations applied to pollen-containing floral structures should be favoured in buzz-pollinating bees. Theoretical work suggests that the rate of pollen release from poricidal anthers is positively related to anther velocity (Buchmann and Hurley, 1978). In addition to increasing the velocity of thoracic vibrations as described above, bees have other behavioural tools at their disposal to increase anther velocity during buzz pollination. The capacity to shake floral structures depends not only on the vibrations produced by the bee’s thorax but also on the characteristics of the flower and on the bee–flower coupling (King, 1993; Arroyo-Correa *et al.*, 2019; de Langre, 2019; Switzer *et al.*, 2019; Vallejo-Marín, 2019; Velilla *et al.*, 2020; Timerman and Barrett, 2021). Bees may benefit from selecting to visit flowers in which they may impose higher accelerations to the anthers and thus increase the rate of pollen removal (e.g. pollen removed per time spent buzzing). For example, a bee of a given size and characteristics may be able to generate a certain maximum thoracic force (f_bee_) determined by both the mass of the bee (m_bee_) and the acceleration (a_bee_) that it can generate using its thoracic power muscles (f_bee_=m_bee_×a_bee_; Fig. 5). When buzzing a flower, the mass of the flower (m_flower_) reduces the acceleration that the bee can produce in the coupled bee–flower system (Fig. 5). Thus, the same bee visiting a flower with larger, heavier stamens should generate lower accelerations than when visiting a relatively smaller flower (Switzer *et al.*, 2019). Because pollen release is proportional to the velocity/acceleration of the vibrations applied to the anthers (De Luca *et al.*, 2013; Vallejo-Marín, 2019), it would be expected that visiting the smaller flower would result in higher rates of pollen removal (Fig. 5). In addition, flower handling, including applying a tight grip to the base of the anthers, and the curling of the bee’s body around the flower may also help in the mechanical transmission of vibrations to the flower (King, 1993). In flowers with multiple anthers that are loosely arranged, bees may change how many anthers are vibrated at a time. Small-sized bees that generate relatively smaller forces compared with large-sized bees may be able to induce higher anther velocities if they manipulate a single or a few anthers at once (Cane and Buchmann, 1989). From the plant perspective, selection to reduce pollen removal by floral visitors may select for flower morphologies with larger stamens (e.g. by increasing anther or connective tissue size). Increased effective mass of the floral structures that need to be vibrated may reduce the acceleration experienced by the anthers for a given force applied by the bee since acceleration is inversely proportional to mass (Fig. 5).

## Conclusions

The mechanistic description of how bees generate and modulate vibrations across different behavioural contexts suggests that, during vibratile pollen collection, bees should be able to fine-tune their floral vibrations while visiting different types of flowers to optimize their pollen collection and energy use. Indeed, empirical studies show that bees adjust the length and number of buzzes in response to pollen availability in poricidal flowers (e.g. Buchmann and Cane, 1989; Vallejo-Marin *et al*., 2009; Papaj *et al.*, 2017; Switzer *et al.*, 2019). However, although observational studies have shown ample variation in other characteristics of bee buzzes across different bee and plant species, to date there is limited experimental evidence of plant-specific adjustment in the frequency and amplitude properties of buzzes produced in different natural and artificial flowers (Corbet and Huang, 2014; Switzer and Combes, 2016; Switzer *et al.*, 2019; Nunes *et al.*, 2021). For instance, the amplitude of floral vibrations is increased when pollen becomes unavailable (Switzer *et al.*, 2019), and the frequency of floral buzzes is slightly reduced (~20 Hz) as individual bees gain experience at manipulating flowers (Morgan *et al.*, 2016; Whitehorn *et al.*, 2017). In a field study comparing different species of buzz-pollinated *Pedicularis*, Corbet and Huang (2014) found that bumblebees applied different floral buzz frequencies to each species. However, the difference was partly explained by assortment of bees to different plant species according to bee size. In contrast, experiments with single bee species in flight cages show no adjustment of buzz frequency to artificial flowers that condition pollen release to specific pre-determined frequencies (Switzer *et al.*, 2019), or to natural flowers with different resonant frequencies (Nunes *et al.*, 2021). These mixed results highlight the need for further studies of floral vibrations across a wider range of bee and plant species. The study of buzz pollination provides a rich field for dissecting how biomechanical, physiological, behavioural, and ecological characteristics of both plants and animals yield an evolutionarily widespread and ecologically important, close interaction between flowers and pollinators.
